# Scapinin, the Protein Phosphatase 1 Binding Protein, Enhances Cell Spreading and Motility by Interacting with the Actin Cytoskeleton

**DOI:** 10.1371/journal.pone.0004247

**Published:** 2009-01-22

**Authors:** Junji Sagara, Toshiaki Arata, Shunichiro Taniguchi

**Affiliations:** 1 Department of Biomedical Laboratory Sciences, School of Health Sciences, Shinshu University, Matsumoto, Nagano, Japan; 2 Department of Molecular Oncology, Graduate School of Medicine, Shinshu University, Matsumoto, Nagano, Japan; 3 Department of Biological Sciences, Graduate School of Science, Osaka University, Toyonaka, Osaka, Japan; University of Birmingham, United Kingdom

## Abstract

Scapinin, also named phactr3, is an actin and protein phosphatase 1 (PP1) binding protein, which is expressed in the adult brain and some tumor cells. At present, the role(s) of scapinin in the brain and tumors are poorly understood. We show that the RPEL-repeat domain of scapinin, which is responsible for its direct interaction with actin, inhibits actin polymerization *in vitro*. Next, we established a Hela cell line, where scapinin expression was induced by tetracycline. In these cells, expression of scapinin stimulated cell spreading and motility. Scapinin was colocalized with actin at the edge of spreading cells. To explore the roles of the RPEL-repeat and PP1-binding domains, we expressed wild-type and mutant scapinins as fusion proteins with green fluorescence protein (GFP) in Cos7 cells. Expression of GFP-scapinin (wild type) also stimulated cell spreading, but mutation in the RPEL-repeat domain abolished both the actin binding and the cell spreading activity. PP1-binding deficient mutants strongly induced cell retraction. Long and branched cytoplasmic processes were developed during the cell retraction. These results suggest that scapinin enhances cell spreading and motility through direct interaction with actin and that PP1 plays a regulatory role in scapinin-induced morphological changes.

## Introduction

The actin cytoskeleton is a vital component of a wide variety of cellular and developmental processes such as cell adhesion, cell migration, cell-cell interaction, and signal transduction [1]–[3]. Cell migration is a critical step in tumor invasion and metastasis, and regulation of this process could lead to therapies for treating cancer [4]. During cell adhesion and migration, the actin cytoskeleton is dynamically reorganized. Various kinds of actin interacting proteins and Rho family GTPases play central roles in actin cytoskeleton reorganization, such as the assembly and disassembly of actin filaments in lamellipodia and filopodia [5]–[7]. In addition, many studies have shown actin to be a component of chromatin remodeling complexes, mRNP complexes, and RNA transcription complexes [8]–[10].

Scapinin was initially identified as a PP1-binding protein that is tightly associated with nuclear non-chromatin structures (the nuclear matrix or nucleoskeleton) in HL-60 cells, a leukemia cell line [11]. Scapinin was down-regulated in HL-60 cells during differentiation induced by all-trans retinoic acid, an anti-tumor drug. EST clone analysis revealed the presence of scapinin gene transcripts in some cancer cells, for example lung and kidney carcinomas [11]. However, in normal adult tissues, scapinin is selectively expressed in the brain. At present, the role(s) of scapinin in the brain and tumors are unknown.

Scapinin is a member of phactr/scapinin family, in which the PP1-binding domain and the RPEL-repeat domain are highly conserved. This family includes four members, phactr1, phactr2, scapinin/phactr3, and phactr4 [12]. PP1 is a major eukaryotic serine/threonine protein phosphatase that regulates diverse cellular processes such as muscle contraction, glycogen metabolism, neuronal signaling, and actin cytoskeleton organization [13]–[15]. The catalytic subunit of PP1 binds to regulatory subunits that are critical for substrate specificity and spatial control of PP1 within the cell. Allen et al. [12] demonstrated that the RPEL-repeat domain of phactr1 is responsible for actin binding. However, it remains unclear whether the RPEL-repeat domain interacts with actin directly or indirectly.

The RPEL-repeat domain is composed of tandem repeats of three RPEL motifs (pfam PF02755) and was initially identified as an actin binding domain in megakaryocytic acute leukemia protein (MAL) [16]. MAL is a transcriptional coactivator for serum response factor (SRF), a transcription factor that activates the mitogen-responsive genes. The dynamics of actin (assembly and disassembly) control SRF activity by regulating the subcellular localization of MAL (cytoplasm or nucleus) [16], [17]. The RPEL-repeat domain of MAL interacts with monomeric actin only. It also works as a sensor for the actin monomer pool. The actin monomer pool is controlled by extracellular growth stimuli such as serum, through Rho family GTPase. Serum-activated Rho GTPase shifts the equilibrium of actin dynamics from the monomeric form to the filamentous form, and consequently MAL loses actin monomers and accumulates in the nucleus.

To explore the function(s) of scapinin, we expressed scapinin in cells and monitored their morphological changes. Scapinin expression stimulated cell spreading and motility. Furthermore, mutation experiments showed that both the RPEL-repeat domain and the PP1-binding domain play crucial roles in these morphological changes. Scapinin was colocalized with actin at the edge of spreading cells. The RPEL-repeat domain of scapinin directly interacts with actin and inhibits actin polymerization *in vitro*. From these results, we propose a role for scapinin as a regulator of actin cytoskeleton structures.

## Methods

### Antibodies and Whole Brain Lysate

Scapinin monoclonal antibody was developed as described [11]. Actin (clone AC-20) and GFP (M2) monoclonal antibodies were purchased from Sigma. Anti-PP1 rabbit antibody was purchased from Santa Cruz Technology Inc. (CA, USA). Human whole brain lysate for SDS-polyacrylamide gel electrophoresis was obtained from Clontech Laboratories Inc. (CA, USA).

### Plasmid Construction and Mutation

The full-length scapinin cDNA was obtained by RT-PCR using a RT primer 5′-AATCTCTATGGCCTGTGGAA-3′, a reverse primer 5′-TCTCTATGGCCTGTGGAATCT-3′, a forward primer 5′-CTGGATGAGATGGACCAAACG-3′, a template poly A^+^ RNA of HL-60 cells, and a high fidelity RNA PCR kit (Takara, Japan). The 1.55 kb RT-PCR product was integrated into *Sma*I site in pKF18k (Takara, Japan) and sequenced. The *Bam*HI/*Eco*RI fragment from pKF18k/scapinin was inserted into *Bgl*II/*Eco*RI sites of pEGFP-c2 (Clontech) or into *Bam*HI/*Eco*RI sites of pcDNA3 (Invitorgen). Deletion and point mutants of pEGFP-c2/scapinin were generated by PCR based mutagenesis as described [11]. The Primers were designed according to the sequence data of scapinin (accession number, AB098521). Mutation in pEGFP-c2-scapinin was confirmed by sequencing. The Expand High-Fidelity DNA polymerase (Roche Molecular Biochemicals) was used for PCR.

### Cell Culture and Transfection

Cos7 and Hela cells were cultured in Dulbecco's modified minimal essential medium (DMEM) containing 10% fetal bovine serum, 100 units/ml penicillin, and 100 µg/ml streptomycin. One day before transfection, cells were seeded on Lab-Tek 8-chambered coverglass or slideglass (Nalge Nunc International). 0.1 µg of pEGFP-c2-scapinin and 0.3 µl of the transfection reagent Fugene 6 (Roche Molecular Biochemicals) were mixed and added to cell cultures (0.25 ml medium) according to the manufacturer's instructions. Transfected cells were cultured in DMEM containing 0.5% fetal bovine serum after transfection. Fluorescent cells were observed in the viable state by using a fluorescent microscope, Axiovert S100 (Carl Zeiss Corporation). In starvation/serum stimulation experiments, the concentration of serum was reduced to 0.5% at 3 hours after transfection, and 10% serum was added to the medium for serum stimulation.

### Conditional Expression of Scapinin in Hela Cells

To establish Hela cell lines, in which scapinin expression is inducible, we used a tetracycline-regulated mammalian expression system, the T-Rex system (Invitrogen Life Technology). In this system, scapinin expression is depressed in the absence of tetracycline and is induced by the addition of tetracycline to the culture medium. We transfected pcDNA6/TR coding repressor proteins for the tetracycline operator to Hela cells and established a cell line that constitutively produced repressors. We then, transfected pcDNA4/TO/myc-His where the full-length scapinin cDNA was inserted between the *Bam*HI and *Eco*RI sites and established in a Hela cell line. We did not use myc- or 6xhistidine-tags in this study, thus any scapinin expressed was untagged. All procedures in establishing cell lines were followed according to the manufacturer's instructions.

### Proliferation Assay

Cell proliferation was measured by an MTT assay. Hela cells established as above were plated at 1×10^4^ cells/ well in 96-well plates and cultured with or without 0.1 µg/ml tetracycline. At each time point, the number of viable cells was assessed by the MTT assay according to the manufacturer's instructions (Promega), and the absorbance was measured at a wavelength of 570 nm with a 96-well plate reader.

### Wound Healing Assay

To measure cell motility, we used a wound healing assay. Hela cells were cultured on 6-well plates (Falcon) until confluent. The confluent monolayer cultures were treated with or without 0.1 µg/ml tetracycline for 4 hours and were then wounded with a straight scratch using a yellow pipette tip. After washing them three times with serum-free DMEM, the wounded monolayer cultures were further incubated with or without 0.1 µg/ml tetracycline in DMEM containing 1% fetal bovine serum. To reduce cell growth, the serum concentration was reduced to 1%.

### Immunofluorescence Staining and Cell Spreading Assay

Scapinin-inducible Hela cells were grown on Lab-Tek 8-chambered coverglass (Nalge Nunc International) and cultured in the presence or absence of 0.1 µg/ml tetracycline to induce scapinin expression. The cells were fixed with 3.7% formaldehyde in phosphate buffered saline for 15 minutes at room temperature, permealized with 0.2% Triton X-100 in PBS for 2 minutes, and incubated with blocking solution (1% bovine serum albumin and 2% fetal bovine serum in PBS) for 15 minutes. The cells were then incubated with 0.2 µg/ml anti-scapinin monoclonal antibody for 1 hour. Anti-scapinin monoclonal antibody was developed as described [13]. After rinsing several times with PBS, the cells were incubated with fluorescein isothiocyanate (FITC)-anti-mouse IgG antibody and rhodamine-phalloidin for 30 minutes. This was rinsed again in PBS, before viewing specimens under a fluorescent microscope. To monitor the effect of scapinin on cell spreading, scapinin-inducible Hela cells grown on dishes were trypsinised with trypsin/EDTA and transferred to Lab-Tek 8-chambered slideglass (Nalge Nunc International). To induce scapinin expression, tetracycline was added to each chamber at a final concentration of 0.1 µg/ml. At time points specified in the figures, cell morphology was photographed randomly under a phase-contrast microscopy and the percentage of adherent cells were determined. We counted over 100 cells per chamber. We used glass slides as opposed to plastic culture dishes because the differences in cell spreading by scapinin expression were more apparent. Parental and control (expressing no scapinin) Hela cells adhere poorly to glassware.

### Confocal Microscopic Observation

Scapinin-inducible Hela cells and GFP-scapinin-expressing Cos7 cells were grown on Lab-Tek 8-chambered coverglass and fixed with 3.7% formaldehyde as described above. In the Hela cells, the scapinin was stained with scapinin monoclonal antibody (the first antibody). Alexa Fluor 488-conjugated goat anti-mouse antibody (Molecular Probes) was used as the secondary antibody. Actin was stained with rhodamine-phalloidin. The localization of scapinin, GFP-scapinin, and actin was observed under a laser scanning microscope LSM 5 EXCITER (Carl Zeiss MicroImaging Inc.).

### Measurement of Cell Area

Scapinin-inducible Hela cells grown on Lab-Tek 8-chambered slideglass were cultured in the presence or absence of tetracycline as described above. The cells were monitored and randomly photographed. After printing the photographs, the cells were cut out with a scalpel, and their weights were determined with a quartz crystal microbalance (Sartorius, Germany). Cos7 cells that expressed GFP or GFP-scapinin were randomly photographed under a fluorescent microscope. Fluorescent Cos7 cells were cut out of the printed photographs and weighed with a quartz crystal microbalance as above. To calculate the cell area (µm^2^) from weights, the ruled 50×50 µ m area (2,500 µm^2^) of a hemocytometer was used as a standard.

### Western Blotting and Densitometric Analysis

Western blotting was performed, and signals were detected by enhanced chemiluminescence method (Amersham) as described previously [11]. Chemiluminescence images were analyzed with LightCapture AE-60-960 (ATTO, Japan).

### GST Pull-Down Assay

The cDNA fragment of Scapinin coding RPEL repeat(s) was amplified by PCR using a specific primer set, to which *EcoR*I (forward primer) or *Sal*I (reverse primer) recognition sequence were added. The cDNA fragment was inserted into *Eco*RI/*Sa*lI sites in pGEX-4T-1 (Amersham Biosciences). GST fusion proteins were produced in *E. coli* BL21 (DE3) pLys and purified with glutathione-sepharose (Amersham Biosciences). Each GST fusion protein was conjugated to CNBr-activated sepharose at a protein concentration of 5 mg/ml bed volume. HL-60 cells (2×10^8^) were lysed in 0.5% Triton X-100/cytoskeleton buffer (10 mM Pipes pH 6.8, 100 mM KCl, 2 mM MgCl_2_, and 0.5 mM EGTA) including protease inhibitor cocktail and sonicated. The cell lysate were separated by centrifugation at 10,000 g for 20 minutes and incubated with GST fusion protein-conjugated sepharose beads for 2 hours. After incubation, the beads were washed twice with 0.5% Triton X-100/cytoskeleton buffer and once with RIPA buffer (0.1% SDS, 0.5% sodium deoxycholate, 1% Nonidet P-40, 50 mM Tris-HCl pH 8.0, 150 mM NaCl). RIPA buffer was used to reduce non-specific binding of actin to sepharose beads. Finally, proteins were extracted from the beads with SDS sample buffer and were subjected to SDS acrylamide gel electrophoresis.

### Co-immunoprecipitation Assay for Actin and PP1 Binding

GFP-scapinin plasmids were transfected into Cos7 cells (∼5×10^5^) grown on 100 mm dishes, and after 24 hours the cells were suspended in 0.5% Triton X-100/cytoskeleton buffer including protease inhibitor cocktail with the aid of sonication. After centrifugation at 10,000 g for 15 mins, the supernatants were incubated with anti-GFP antibody for 3 hours. Immunocomplexes were collected with protein G-agarose beads that had been washed twice with 0.5% Triton X-100/cytoskeleton buffer. For the actin binding assay, after washing with 0.5% Triton X-100/cytoskeleton buffer, protein G-agarose beads were washed once with RIPA buffer to reduce the non-specific binding of actin to agarose beads. Immunocomplexes were subjected to Western blotting with anti-scapinin monoclonal antibody, anti-actin monoclonal antibody, or anti-PP1 polyclonal antibodies.

### Actin Polymerization

Actin polymerization was quantified in the presence of various molar ratio of RPEL-GST. Skeletal muscle actin was purified from dry rabbit muscle, and actin polymerization was measured by an ultracentrifugation assay as described [18]. To polymerize actin, a solution consisting of G-actin and GST-RPEL was adjusted to produces final concentrations of 100 mM KCl, 2 mM MgCl2, and 10 mM Tris-HcCl (pH 7.8) and incubated at 20°C for 60 min. After sedimentation at 130,000 g for 15 min, the supernatant and pellet were subjected to SDS-PAGE. The peak areas of actin bands in the supernatant and pellet were measured by a densitometer (Fuji-Riken), and the fraction of depolymerized actin (Fb) was calculated as Fb = S/(S+P).

The apparent affinity of RPEL-GST for actin was determined by exploiting its ability to inhibit actin polymerization. The dissociation constants (Kd) and number of RPEL binding sites of actin (n) were calculated by nonlinear regression in Igor Pro software (WaveMetrics, Oregon) using the following equation:
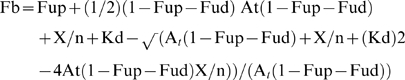
where A_t_ and X are the total actin and total GST-RPEL concentrations, respectively. Fup and Fud indicate the fractions of contaminated (denatured) actins that were unable to polymerize even without GST-RPEL and unable to bind to GST-RPEL and depolymerize, respectively. At and Fup are known values, and Fb is a function of X.

## Results

### Domain Structure of Scapinin

**Figure 1:**
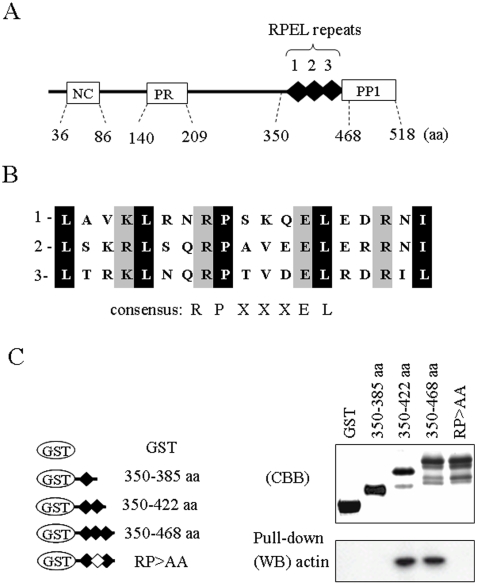
Interaction of the RPEL repeats of scapinin with cellular actin. (*A*) The primary structure of scapinin. The N-terminal region that is conserved in the scapinin/phactr family (NC), the proline-rich region (PR), the three tandem repeats of PREL motifs (RPEL repeats 1, 2, and 3), and the PP1-binding domains (PP1) are indicated. In addition to the N-terminal conserved region (NC), the RPEL-repeat and PP1-binding domains are highly conserved in the scapinin/phactr family. (*B*) Three RPEL motifs are compared. The RPEL motif (pfam PF02755) is named according to the conserved amino acid sequence, RPxxxEL. (*C*) Interactions between the RPEL repeats of scapinin and cellular actin. The RPEL repeats of scapinin were produced as fusion proteins with glutathione S-transferase (GST) in *E. Coli* as illustrated. In the RP>AA mutant, both the arginine and proline residues in the second motif of the RPEL repeats were substituted with alanine residues. Purified GST-RPEL constructs were separated by SDS-polyacrylamide gel electrophoresis and stained with coomassie brilliant blue (CBB). GST-RPEL constructs were covalently conjugated to CNBr-agarose beads (5 mg protein/ml bed volume) and subjected to a pull-down assay with HL-60 cell lysates (see ‘Methods’). Actin in the pull-down-samples was determined by Western blotting (WB) with anti-actin antibody. The GST-RPEL construct (350–465 aa) contained substantial amounts of cleaved products (see ‘Results’).

Fig. 1 illustrates the domain structure of scapinin. The C-terminal region was identified as a PP1-binding domain [11]. The RPEL-repeat domain is composed of tandem repeats of three RPEL motifs. The PP1-binding domain, RPEL-repeat domain, and N-terminal region are highly conserved in the phactr/scapinin family, suggesting important physiological roles for these domains.

### RPEL-repeat Domain and Actin Binding

We applied the yeast two-hybrid method to determine scapinin-binding proteins. We have previously determined PP1 as a binding partner with this approach. After further analysis using the C-terminal region (300–518 aa) as a bite, we obtained eight independent clones that coded for β-actin cDNA (data not shown). Three RPEL motifs are perfect fits for the consensus sequence, RPxxxEL, of the RPEL motif (Fig. 1B). Deletion mutant experiments demonstrated that phactr1 binds with actin through the RPEL-repeat domain [12]. However, it is unclear whether the RPEL-repeat domain interacts with actin directly or indirectly.

Several GST-RPEL constructs (Fig. 1C) were conjugated to agarose beads and subjected to an *in vitro* pull-down assay with HL-60 cell lysates (Fig. 1C). Cell lysates and GST-RPEL beads were incubated in 0.5% Triton X-100/cytoskeleton buffer (10 mM Pipes pH 6.8, 100 mM KCl, 2 mM MgCl_2_, and 0.5 mM EGTA). To reduce the non-specific binding of actin to agarose beads, GST-RPEL conjugated agarose beads (pull-down samples) were washed twice with 0.5% Triton X-100/cytoskeleton buffer and once with RIPA buffer (0.1% SDS, 0.5% sodium deoxycholate, 1% Nonidet P-40, 50 mM Tris-HCl pH 8.0, 150 mM NaCl). Without a stringent wash with RIPA buffer, substantial amounts of actin were nonspecifically co-precipitated with the agarose beads. Scapinin-RPEL constructs of 350–468 aa and 350–422 aa bound with actin *in vitro*, but a single RPEL motif construct (350–385 aa) showed no actin-binding activity (Fig. 1C).

It is documented that MAL loses its actin-binding activity after double mutations at two arginine residues (RR33/77DD) or two proline residues (PP34/78AA) of the PREL-repeat domain [16]. Guettler et al. [19] showed that single RPEL mutations (R>A or P>A) of the MAL-RPEL-repeat domain affect its subcellular localization and transcriptional cofactor activity, demonstrating that the integrity of all three RPEL motifs is required for MAL regulation. Next, we introduced mutations into the second motif of the scapinin-RPEL-repeat domain (RP>AA mutant). The RP>AA mutant exhibited no actin binding activity (Fig. 1C).

As the GST-RPEL construct of 350–465 aa is highly insoluble, only ∼30% of them were recovered from *E. Coli*, and the purified samples included substantial amounts of cleaved polypeptides (Fig. 1C). Furthermore, more than half of the purified polypeptides were precipitated during dialysis against actin polymerizing buffer. At present, the reason for the insolubility of this construct is unknown.

### Inhibition of Actin Polymerization by the RPEL Repeats of Scapinin *in vitro*

**Figure 2:**
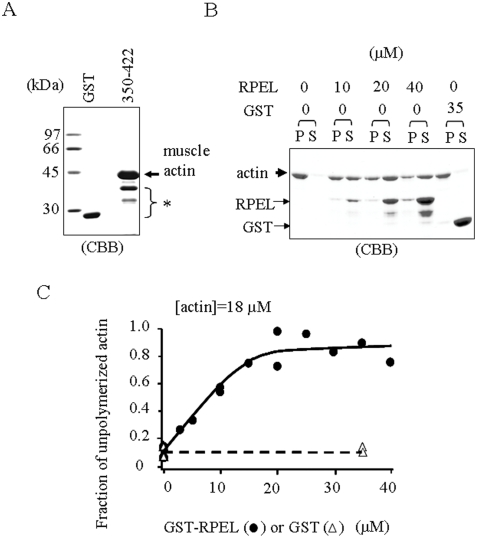
The RPEL repeats of scapinin interact with purified skeletal muscle actin and inhibit actin polymerization *in vitro*. (*A*) GST or GST-RPEL repeats (350–422 aa) were covalently conjugated to CNBr-agarose beads (as in Figure 1C) and were incubated with purified skeletal muscle actin. After washing with RIPA buffer (0.1% SDS, 0.5% sodium deoxycholate, 1% Nonidet P-40, 50 mM Tris-HCl pH 8.0, 150 mM NaCl), bound proteins were eluted with SDS sample buffer, separated by SDS-polyacrylamide gel electrophoresis, and stained with coomassie brilliant blue (CBB). GST and GST-RPEL proteins (shown by a asterisk) were partly released from the beads by elution with SDS sample buffer. (*B*) Inhibition of actin polymerization by the RPEL repeats. Skeletal muscle actin (18 µM) was incubated with GST- RPEL repeats (350–422 aa) or GST at the indicated concentrations to polymerize at room temperature for 30 minutes, and then filamentous actin (P) and monomeric actin (S) were separated by ultracentrifugation. Aliquots were analyzed by SDS polyacrylamide gel electrophoresis and stained with coomassie brilliant blue (CBB). Since the contamination of supernatants in the pellet fraction was technically inevitable, a small portion of GST-RPEL construct was seen in the pellet. (*C*) The density of each actin band was measured by a densitometer and plotted.

The GST-RPEL construct of MAL interacts with purified skeletal muscle actin and inhibits actin polymerization *in vitro* [20]. Next, we tested whether the GST-RPEL construct of scapinin has the same properties as the MAL construct. The GST-RPEL construct of 350–468 aa self-aggregated during dialysis against actin polymerizing buffer, and the purified samples included substantial amounts of cleaved polypeptide (see above). Thus, we used the GST-RPEL construct of 350–422 aa for further studies. The GST-RPEL construct of 350–422 aa bound to purified skeletal muscle actin (Fig. 2A), demonstrating that the RPEL-repeat domain of scapinin directly interacts with actin without additional accessory proteins.

Next, we examined the effect of the GST-RPEL construct on actin polymerization using an ultracentrifugation method [18]. As the GST-RPEL construct was increased, filamentous actin (the pellet fraction) was reduced, demonstrating that the RPEL repeats inhibit actin polymerization (Fig. 2B). Although a small amount of GST-RPEL polypeptides were seen in the pellet fraction (Fig. 2B), this was due to contamination of the soluble fraction as it is difficult to completely remove soluble proteins from the pellet fraction after ultracentrifugation.

Actin monomer binding proteins such as DNase I, gelsolin, profilin, cofilin, and thymosins induce the depolymerization of actin in vitro. We can assume that the amount of GST-RPEL necessary for depolymerization is equal to that of GST-RPEL bound to actin monomer [21]. Posern et al. [20] determined the dissociation constant of MAL-RPEL-actin complex from a titration with MAL-RPEL for the fluorescence intensity of pyrene-labeled actin, which is an indicator of depolymerization. According to this report, we have determined the apparent dissociation constant of scapinin-RPEL–actin complex from the titration with scapinin-RPEL for the fraction of depolymerized actin (Fig. 2C). Increasing the scapinin-RPEL led to saturation of actin depolymerization at a stoichiometric concentration. The data were consistent with a stoichiometry for scapinin-RPEL to actin of 1.21±0.16 and an apparent dissociation constant of 0.018±0.26 µM (±uncertainty of the fit). These results suggest that the RPEL repeats (359–422 aa) of scapinin interact with actin monomers, but not with actin filament.

Recently Guettler et al. [19] showed that single RPEL motifs (RPEL1, RPEL2, RPEL3) of the MAL-RPEL-repeat domain interact with actin. RPEL1, RPEL2, and RPEL3 of MAL bind with actin with apparent dissociation constants of ∼5.4 µM, ∼2.3 µM, and ∼18.8 µM, respectively. However, a single RPEL motif (350–385 aa) of scapinin showed no actin binding activity (Fig. 1C). A stringent washing step (RIPA buffer) was included in our binding assay to reduce non-specific binding of actin to agarose beads (Fig. 1C). Thus, the discrepancy may due to differences in the assay conditions.

### Stimulation of Cell Spreading and Motility by Scapinin Expression

**Figure 3:**
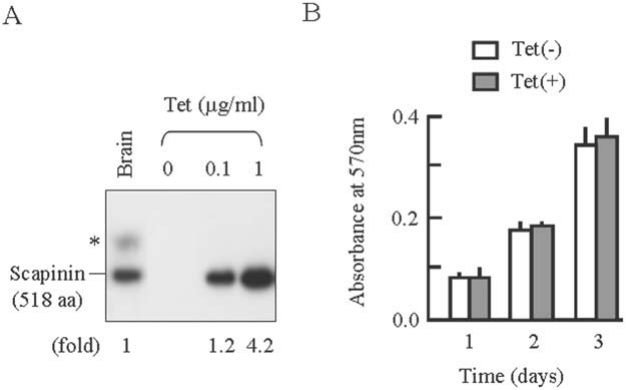
Induction of scapinin expression in Hela cells and its effect on cell proliferation. (*A*) Tetracycline-induced expression of scapinin in Hela cells. Hela cells were cultured with tetracycline at the indicated concentrations for 24 hours. Cell lysates were separated by SDD-polyacrylamide gel electrophoresis, and scapinin expression levels were measured with Western blotting using anti-scapinin monoclonal antibody. Human whole brain lysate (Clontech Laboratories Inc.) was also loaded. Twenty microgram proteins were applied to each lane. Scapinin expression levels of Hela cells were expressed as folds against the whole brain lysate. (*C*) The effect of scapinin on the proliferation of Hela cells. Hela cells were plated at 1×10^4^ cells/ well in 96-well plates and cultured with (open bars) or without (closed bars) 0.1 µg/ml tetracycline. At each time point, the number of viable cells was assessed by an MTT assay, and the absorbance was measured at a wavelength of 570 nm with a 96-well plate reader. Data are expressed as mean±SEM of four separate experiments.

For comprehensive studies, we established a Hela cell line in which scapinin expression was induced by the addition of tetracycline. Scapinin expression levels in Hela cells at 0.1 µg/ml teracycline were almost comparable to those in the human brain (Fig. 3A). In the human brain, an additional polypeptide (shown by an asterisk) is seen in Western blots with anti-scapinin antibody. This additional polypeptide seems to be a variant form (559 aa) of scapinin (accession number, NP_542403). We obtained essentially the same results for cell proliferation, cell spreading, and cell motility at both 0.1 and 1 µg/ml tetracycline (see below). Data from 0.1 µg/ml tetracycline are presented here unless otherwise stated.

We examined the effect of scapinin on cell proliferation. Scapinin expression at 0.1 µg/ml tetracycline did not significantly affect the growth rate of Hela cells (Fig. 3B). Even at 1 µg/ml tetracycline, no significant differences were detected between scapinin-expressing and non-expressing cells. This result demonstrates that scapinin expression neither stimulates nor inhibits cell proliferation in Hela cells.

**Figure 4:**
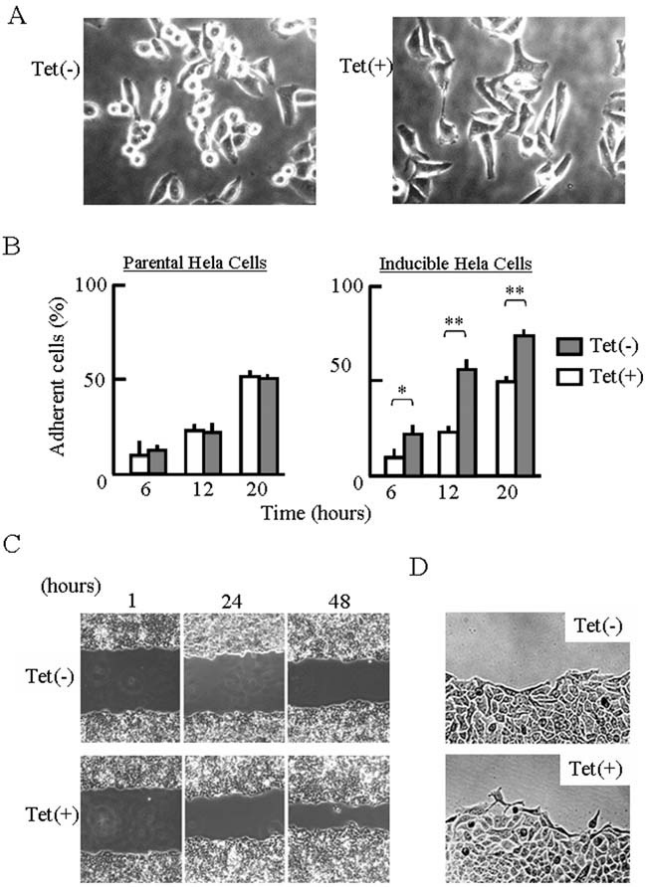
Enhancement of cell spreading and motility by scapinin. (*A*) Enhancement of cell spreading by scapinin. Inducible Hela cells were seeded onto an 8-chambered glass-slide and then cultured with (Tet+) or without (Tet−) 0.1 µg/ml tetracycline. Cell morphology was monitored with a light microscope and photographed at 12 hours. (*B*) Inducible Hela cells were treated as in (*A*), and cell morphology was monitored at specified times. Adherent and non-adherent cells were counted under a microscope. Data are expressed as mean±SED from four independent experiments. Student's t test: *: P<0.05; **: P<0.01. The parental Hela cells were also cultured with (Tet+) or without (Tet−) of tetracycline, and cell morphology was monitored. (*C*) Enhancement of cell motility by scapinin. To measure cell motility, we used a wound healing assay. Hela cells were cultured on 6-well plates until confluence. The confluent monolayer cultures were treated with (Tet+) or without (Tet−) 0.1 µg/ml tetracycline for 4 hours and were then wounded with a straight scratch using a yellow pipette tip. After washing them three times with serum-free DMEM, the wounded monolayer cultures were further incubated (Tet+) with or without (Tet−) 0.1 µg/ml tetracycline in DMEM containing 1% fetal bovine serum. To reduce cell growth, the serum concentration was reduced to 1%. At 1, 24, and 48 hours after the wounding, the cell monolayer was photographed. The front of cell migration at 24 hours was shown in (*D*).

Cell spreading was significantly enhanced by expression of scapinin (Fig. 4A). Scapinin-expressing cells adhered to glassslides faster than non-expressing cells (Fig. 4B). Next we measured cell area of adherent cells at 20 hours. To focus on adherent cells, rounded cells were excluded from this measurement. Cell area of adherent cells of non-expressing (Tet−) and scapinin-expressing (Tet+) cells were 586±53 µm^2^ and 883±95 µm^2^ (mean±SD, n = 50), respectively. Cell spreading of parental Hela cells was not enhanced by tetracycline treatment (Fig. 4B). Notably, scapinin-expressing cells frequently exhibited elongated shapes that were rarely seen in the parental Hela cells used in this study.

We assessed the effect of scapinin expression on cell motility using a wound healing assay. The expression of scapinin markedly enhanced cell motility (Fig. 4C). To reduce cell growth, we performed the wound healing assay with 1% fetal bovine serum. It is notable that whereas non-expressing cells (Tet−) collectively moved, scapinin-expressing cells (Tet+) migrated as single cells (Figure 4D).The parental Hela cells used in this study moved in sheet-like structures like the non-expressing cells. The cell motility of the parental cells was not enhanced by tetracycline treatment.

### Colocalization of Scapinin with Actin at the Edge of Spreading Cells

**Figure 5:**
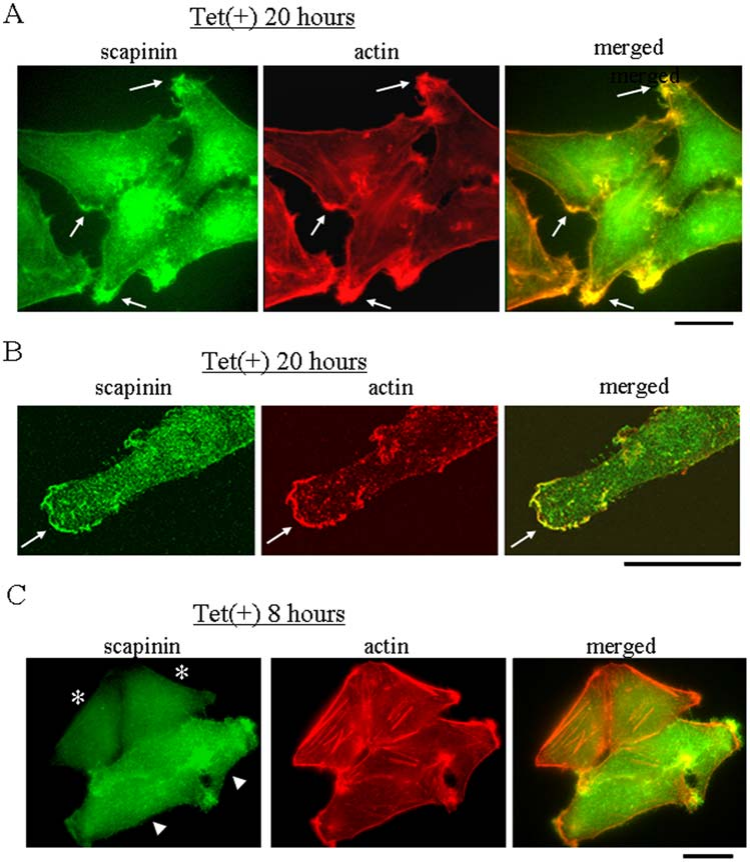
Distribution of scapinin and actin in Hela cells. (*A*) Hela cells grown on glass coverslips were cultured in the presence (Tet+) or absence (Tet−) of 0.1 µg/ml tetracycline and were then fixed at 20 hours. After permeabilization, the distribution of scapinin and the actin cytoskeleton were visualized by staining with anti-scapinin antibody (green) and rhodamine-phalloidin (red), respectively. Scapinin and actin are colocalized (arrows). (*B*) Confocal microscopic observation. Hela cells were cultured in the presence of 0.1 µg/ml tetracycline for 20 hours and were then stained with anti-scapinin antibody (green) and rhodamine-phalloidin (red) as in (*A*). Scapinin and actin are colocalized (arrows). Bar: 20 µm. (*C*) Absence of scapinin in actin stress fibers. Hela cells grown on a glass coverslip were cultured in the presence of 0.1 µg/ml tetracycline and were then fixed at 8 hours. The distribution of scapinin and the actin cytoskeleton were visualized by staining with anti-scapinin antibody (green) and rhodamine-phalloidin (red), respectively as (*A*). There are four cells; two cells express low levels of scapinin (asterisks), and the other two cells express high levels of scapinin (arrow heads). Bar: 20 µm.

Immunostaining showed that scapinin and actin were mostly colocalized at the edges of spreading cells (Fig. 5A). Confocal microscopic observation also showed colocalization of scapinin and actin (Fig. 5B). Shortly after tetracycline-treatment, when scapinin expression levels were low, actin stress fibers were seen in some cells, but as scapinin expression levels were increased, the number of actin stress fibers was reduced (Fig. 5C). In addition, scapinin staining was not seen in actin stress fibers (Fig. 5C). These results suggest that scapinin promotes cell spreading and motility through reorganization of the actin cytoskeleton.

Scapinin was predominantly distributed in the cytoplasm at all serum concentrations tested (0.5∼15%). MAL translocates from the cytoplasm to the nucleus upon serum stimulation (15% FBS) after starvation (0.5% FBS) [16]. In contrast to MAL, scapinin exhibited no significant cytoplasm-to-nucleus translocation upon serum stimulation after starvation.

### GFP-scapinin Induced Cell Spreading in Cos7 Cells

**Figure 6:**
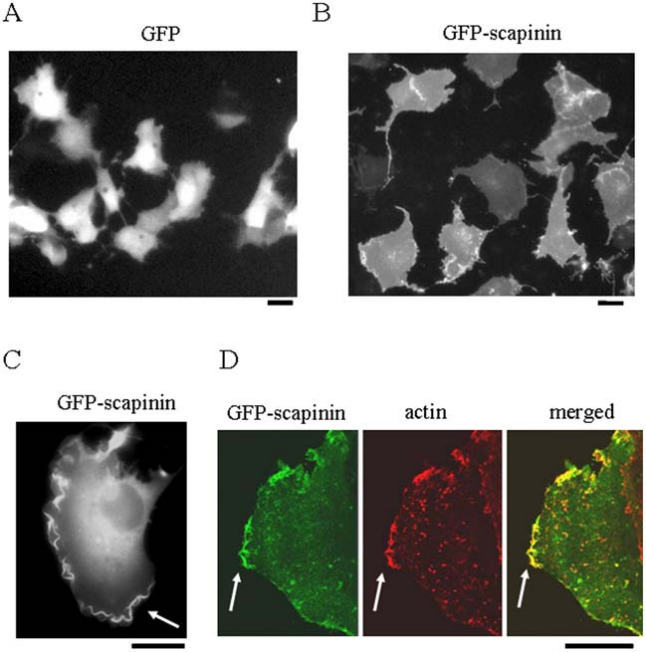
GFP-scapinin-induced morphological changes in Cos7 cells. pEGFP empty vectors (*A*) and pEGFP-scapinin (*B*) were transfected into Cos7 cells, and the cells were cultured for 16 hours. The cell morphology and the distribution of GFP-scapinin were monitored with fluorescent microscopy and photographed. (*C*) The distribution of GFP-scapinin in ruffles. (*D*) Colocalization of scapinin and actin in ruffles. Cos7 cells expressing GFP-scapinin were fixed at 16 hours and then stained with rhodamine-phalloidin (red) to visualize actin. Localization of GFP-scapinin (green) and actin (red) was observed with a confocal microscopy. Ruffles were shown by arrows. Bars: 20 µm.

The expression of proteins fused to GFP has made it possible to localize and study the dynamics of living cells. Expression of GFP-scapinin (wild type) also induced cell spreading in Cos7 cells (Fig. 6A and B). Cell area of adherent cells of GFP-expressing (Tet−) and GFP-scapinin-expressing (Tet+) cells were 777±73 µm^2^ and 1576±221 µm^2^ (mean±SD, n = 50) respectively. In Cos7 cells, GFP-scapinin was predominantly distributed throughout cells. Notably, GFP-scapinin was frequently distributed in membrane ruffle-like structures (Fig. 6C). Confocal microscopy revealed colocalization of GFP-scapinin and actin in ruffle-like structures (Fig. 6D). This result indicated that GFP-scapinin is localized in actin-based ruffles. In only a small number of cells (less than 15%), distinctive GFP-scapinin signals were seen in the nucleus.

### The Roles of the RPEL-repeat and PP1-Binding Domains in Cell Spreading

**Figure 7:**
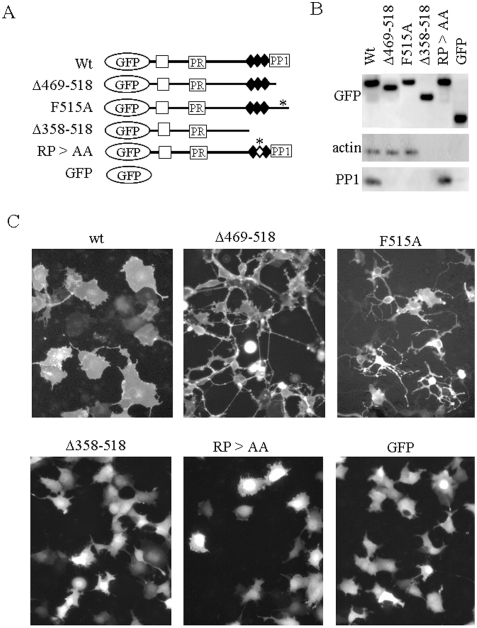
The roles of the RPEL-repeat and PP1-binding domains in scapinin-cell spreading. (*A*) The pEGFP-scapinin mutants used in this study are illustrated. (*B*) Each construct shown in (*A*) was transfected into Cos7 cells. The cells were cultured for 24 hours and lysed in 0.5% Triton X-100/cytoskeleton buffer at 24 hours. GFP-scapinins were immunoprecipitated with anti-GFP antibody, and immunocomplexes were collected with protein A-agarose beads, washed, and subjected to Western blotting with anti-scapinin monoclonal antibody, anti-actin monoclonal antibody, and anti-PP1 polyclonal antibodies, respectively. (*C*) Each pEGFP-scapinin mutant was transfected into Cos7 cells. Cells were cultured for 16 hours and then monitored under a fluorescent microscope and photographed. Bar: 20 µm.

Two approaches using a transient expression system (Fig. 6) and a tetracycline-induced expression system (Fig. 4) gave essentially identical results with respect to cell spreading. To explore the roles of the RPEL-repeat and PP1-binding domains, we made actin-binding and PP1-binding deficient mutants and tested their ability to stimulate cell spreading. The deletion and point mutants used in this study are shown in Fig. 7A, and interactions of each GFP-scapinin mutant with endogenous actin and PP1 were examined by a co-immunoprecipitation assay (Fig. 7B).

A mutant without the PP1-binding domain (Δ469–518) induced cell rounding or shrinkage instead of cell spreading. Notably, cell rounding and shrinkage were accompanied by development of long and branched cytoplasmic processes. Allen et al. [12] demonstrated that replacement of phenylalanine residue (F) with alanine (A) in this C-terminal region impairs the PP1-binding activity of Phactr1. We mutated the phenylalanine residue of scapinin essential for PP1 binding to alanine (F515A). This point mutant showed essentially the same results as the Δ469–518 mutant (Fig. 7C). These PP1-binding deficient mutants retained interactions with endogenous actin (Fig. 7B). These results suggest that the PP1-binding domain plays a regulatory role in the morphology changing-activities of scapinin.

**Figure 8:**
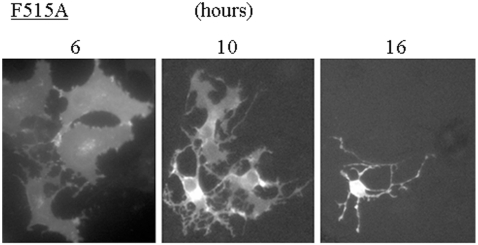
Time course of morphological changes of Cos7 cells induced by a PP1-binding deficient mutant of GFP-scapinin (F515A). After transfection of pEGFP-scapinin F515A mutant, morphological changes of Cos7 cells were monitored and photographed at indicated times under a fluorescence microscopy. Bar: 20 µm.

Time course of morphological changes induced by a PP1-binding deficient mutant of F515A showed that, at early times (6 hours) after transfection, some cells expressing F515A mutant showed spread morphologies like wild-type scapinin expressing cells, but they promptly shrank to show rounded morphologies with long and branched cytoplasmic processes (Fig. 8). We obtained the same results with Δ469–518 mutant. PP1-binding deficient mutants may strongly induce cell retraction although they retain the cell spreading activity.

Further deletion of the C-terminal region including the three RPEL motifs (Δ359–518) completely abolished the morphology-changing activities (Fig. 7C). Δ359–518 deletion mutants abolished both actin and PP1 binding activities (Fig. 7B)

As shown in Fig. 1C, double mutations (RP>AA) in the second RPEL motif of the RPEL-repeat domain abolished actin-binding *in vitro*. Then, we introduced mutations into the second motif of the RPEL repeat, the RP>AA mutant, in which both arginine (407) and proline (408) residues have been replaced with alanine (Fig. 7B). As with the Δ359–518 deletion mutant, the RP>AA mutant exhibited no morphology-changing activities (neither cell spreading nor cell rounding) in Cos7 cells. Accordingly, the RPEL-repeat domain and its actin-binding activity are essential for morphological changes.

## Discussion

Actin cytoskeleton dynamics play essential roles in cell adhesion, cell motility, and cell morphology. Tetracycline-induced expression of scapinin in Hela cells enhances cell spreading and motility at expression levels comparable to those in the human brain (Fig. 3A and 4). In Hela cells, scapinin colocalizes with actin at the edge (maybe the lamellipodiim, ruffle, or filopodium) of spreading cells (Fig. 5). Notably, actin stress fibers were seen in cells expressing lower levels of scapinin, but their number was decreased in cells expressing higher levels of scapinin (Fig. 5). In addition, scapinin-staining was hardly seen in actin stress fibers (Fig. 5). It seems that scapinin expression reduces the number of actin stress fibers and increases the amount of peripheral actin. Mutations in the RPEL-repeat domain abolished both the actin-binding and morphology-changing activities of scapinin (Fig. 7). These results demonstrate that scapinin can modulate actin cytoskeleton structures and thereby enhance cell spreading and motility.

The RPEL-repeat domain of scapinin interacts with purified skeletal muscle actin without any accessory proteins (Fig. 2A). Mutations in the RPEL-repeat domain abolished both the actin-binding and morphology-changing activities of scapinin (Fig. 1C and 7). These results clearly demonstrate that direct interactions with actin through the RPEL-repeat domain are essential for scapinin-induced morphological changes.

It is known that the RPEL-repeat domain of MAL interacts with actin monomers, but not with actin filaments [16]. The GST-RPEL construct of MAL inhibits actin polymerization *in vitro*, supporting the fact that the RPEL-repeated sequence is an actin-monomer-binding motif [20]. The consensus amino acid sequence of RPEL motifs , RPxxxEL, is conserved in the scapinin-RPEL-repeat domain (Fig. 1B). Our *in vitro* study demonstrated that the GST-RPEL construct of scapinin inhibits actin polymerization like that of MAL (Fig. 2B and C). This result suggests that the RPEL-repeat domain of scapinin like MAL may interact with actin monomers, but not with actin filaments. Further studies are required to determine the exact mode of actin-scapinin interactions.

Various kinds of actin-monomer-binding proteins play regulatory roles in actin dynamics, i.e., disassembly and assembly, and thereby govern cell adhesion, cell motility, and cell morphology [22]. For example, although profilin and ADF/cofilin were initially identified as an actin-monomer-sequestering protein and an actin-depolymerizing factor respectively, it is now established that they play central roles in directional actin-filament growth, or ‘treadmilling’ as it is also known [5]–[7]. Wiskott-Aldridge syndrome protein (WASP) homology domain 2 (WH2) is a small and widespread actin-monomer-binding motif. WH2 has versatile regulatory functions in actin dynamics [23], [24]. Among WH2 containing proteins, whereas β-thymosins sequester actin monomers during polymerization, WASP family proteins (WASP, N-WASP, and WAVE) activate Arp2/3-dependent actin nucleation and branching in respond to signals mediated by Rho-family GTPase [25]–[27]. WASP and N-WASP interact with Arp2/3 complex through the C-terminal acidic region. Further biochemical and structural studies are required to determine the exact mode of actin-scapinin interaction by which scapinin enhances cell spreading and motility. However, we do not exclude the possibility that scapinin partly modulates cell morphology at the transcriptional level, increasing or decreasing particular proteins to regulate actin dynamics.

In contrast to scapinin, MAL induces no cell morphological changes after the transfection of expression plasmids into cells (personal communication, Dr. Treisman R.). Favot et al. [28] reported that, when phactr/scapinn family proteins (they named them PPEL proteins) were expressed as GFP fusion proteins in cells, all four family members modified the shape of cells so that a rough appearance at the edges of cells and various lengths of hair-like cytoplasmic extensions were observed. In addition, they also reported that all four members were predominantly distributed in the cytoplasm. Their results are in good agreement with ours and indicate that the cell morphology changing activity is a common feature of all four members of the phactr/scapinin family.

This study suggests that scapinin can enhance cell spreading and motility through interactions with actin cytoskeletons. EST clone analysis demonstrated the presence of scapinin gene transcripts in some tumors cells such as lung and kidney carcinomas. Cell migration is a critical step in tumor invasion and metastasis, and regulation of this process will lead to therapies for treating cancer [4]. Further studies on the expression profile of scapinin in various types of tumor cells and the relationship of scapinin expression levels with malignancy are necessary.

PP1 is a major eukaryotic serine/threonine protein phosphatase that regulates diverse cellular processes such as muscle contraction, glycogen metabolism, neuronal signaling, and actin cytoskeleton organization [13]–[15]. The catalytic subunit of PP1 binds to regulatory subunits that are critical for substrate specificity and spatial control of PP1 within the cell. PP1-binding deficient mutants (Δ469–518 and F515A) induced cell rounding with extensive development of long cytoplasmic processes instead of cell spreading (Fig. 7). This result demonstrates that PP1 plays a regulatory role in scapinin-induced morphological changes.

It was reported that the *humpty dumpty* mouse mutant phenotype with failure to close the neural tube and optic fissure is caused by a missense mutation in phacrt4 (a member of the phactr/scapinin family), and interestingly, the missense mutation specifically disrupts binding to PP1 [29]. This demonstrates that the PP1-binding activity of phactr4 is critical for its physiological role in embryogenesis. Our data also demonstrate that the PP1-binding activity of scapinin is critical for the biological activity of scapinin (Fig. 7).

The *humpty* embryos display elevated proliferation and abnormally phosphorylated, inactive PP1, resulting in RB hyperphosphorylation and derepression of E2F targets. We initially identified scapinin as a component of the nuclear nonchromatin structures (nuclear matrix or nucleoskeleton) in HL-60 cells and found that scapinin is down-regulated during differentiation induced by all-trans retinoic acid treatment (an anti-tumor drug). Recently numerous studies have established the presence of actin in the nucleus and have shown that actin is involved diversely in nuclear activities including gene transcription, chromatin remodeling, and nucleocytoplasmic trafficking [8]–[10], [17]. In addition to actin, actin binding and adhesion proteins are present in the nucleus and modulate nuclear functions [30]. We currently hypothesize dual roles for scapinin in the cytoplasm and nucleus.

The *scapinin/phactr3* gene has four leader exons, implying that regulation of its gene expression may be complicated [31]. Down-regulation of gene expression by RNAi is a powerful tool for studying the physiological roles of many genes. Our attempts to explore the functions of scapinin with RNAi have not yet succeeded. The presence of multiple leader exons in the *scapinin/phactr3* gene may make it difficult. Further genetic studies are necessary to determine the roles of scapinin in cell proliferation and morphogenesis of normal neuronal tissues.
